# Evaluating the role of maladaptive personality traits in schema therapy and cognitive behavioural therapy for depression

**DOI:** 10.1017/S0033291722001209

**Published:** 2023-07

**Authors:** Katharina Rek, Nils Kappelmann, Johannes Zimmermann, Martin Rein, Samy Egli, Johannes Kopf-Beck

**Affiliations:** 1 Max Planck Institute of Psychiatry, Munich, Germany; 2 Department of Psychology, University of Kassel, Kassel, Germany; 3 Department of Research in Translational Psychiatry, Max Planck Institute of Psychiatry, Munich, Germany; 4 International Max Planck Research School for Translational Psychiatry (IMPRS-TP), Munich, Germany; 5 Oberberg Tagesklinik, Munich, Germany; 6 Department of Psychology, LMU Munich, Munich, Germany

**Keywords:** Cognitive behaviour therapy, depression, maladaptive personality traits, outcome prediction, personality change, precision psychiatry, schema therapy

## Abstract

**Background:**

Advancements in the treatment of depression are pivotal due to high levels of non-response and relapse. This study evaluated the role of personality pathology in the treatment of depression by testing whether maladaptive personality traits (1) predict changes in depression over treatment or *vice versa*, (2) change themselves over treatment, (3) change differentially depending on treatment with schema therapy (ST) or cognitive behavioural therapy (CBT), and (4) moderate the effectiveness of these treatments.

**Methods:**

We included 193 depressed inpatients (53.4% women, *M*_age_ = 42.9, SD = 13.4) participating in an assessor-blind randomized clinical trial and receiving a 7-week course of ST or CBT. The research questions were addressed using multiple indicator latent change score models as well as multigroup structural equation models implemented in EffectLiteR.

**Results:**

Maladaptive traits did not predict changes in depressive symptoms at post-treatment, or *vice versa*. However, maladaptive trait domains decreased over treatment (standardized Δ*μ* range: −0.38 to −0.89), irrespective of treatment with ST or CBT. Maladaptive traits at baseline did not moderate the effectiveness of these treatments.

**Conclusions:**

Self-reported maladaptive personality traits can change during treatment of depression, but may have limited prognostic or prescriptive value, at least in the context of ST or CBT. These results need to be replicated using follow-up data, larger and more diverse samples, and informant-rated measures of personality pathology.

## Introduction

With around 300 million affected individuals worldwide, major depressive disorder (MDD) is a highly prevalent and debilitating mental illness not only for those that are affected, but also for society at large (Kessler et al., 2009). Even though psychotherapy and anti-depressant medication have been found to improve depressive symptom severity as monotherapy or in combination (Cuijpers et al., 2020), treatment resistance and relapse rates remain high (Holtzheimer & Mayberg, 2011; Rush et al., 2006). To better understand the challenges and barriers to successful depression treatment, this study aimed to explore the role of personality pathology. Previous studies found that around 45% of patients with MDD suffer from comorbid personality disorders (PDs; Friborg et al., 2014) and MDD is associated with a distinct profile of personality traits including lower emotional stability, extraversion, and conscientiousness (Hakulinen et al., 2015; Kotov, Gamez, Schmidt, & Watson, 2010). However, patients with MDD are also highly heterogeneous in their pattern of personality problems (Cain et al., 2012; Simon, Cain, Wallner Samstag, Meehan, & Muran, 2015), which could be a potential indicator of differential trajectories of change in depression treatments. In the following, based on data from an randomized controlled trial (RCT) comparing two different psychotherapeutic treatments in depressed inpatients (*N* = 193), we investigated (1) whether maladaptive personality traits are associated with changes in depressive symptoms and/or *vice versa*, (2) whether maladaptive personality traits change over the course of treatment, (3) if this change depends on the type of treatment, and (4) if maladaptive personality traits moderate the effectiveness of different treatment types. Our results may inform clinical decisions on the suitability of specific interventions for the individual patient.

Personality pathology may be a promising candidate for treatment response prediction in depression. Based on meta-analytic evidence from prospective case series and RCTs, Newton-Howes et al. (2014) showed that comorbid PDs doubled the odds of poor treatment outcomes in depressed patients regardless of the type of treatment. A more recent meta-analysis restricted to RCTs of cognitive behavioural therapy (CBT) reported similar results overall. However, the adverse impact of PDs on treatment course of depression diminished in subgroup analyses that only included high-quality studies or when adjusting meta-analytic effect estimates for baseline depression severity (Banyard, Behn, & Delgadillo, 2021). Therefore, the predictive utility of personality pathology in treatments of MDD remains unclear. It also remains unclear whether and to what extent pre-treatment depressive symptom levels influence the malleability of personality pathology over treatment itself as suggested by Hellerstein et al. (2010). This would indicate a bi-directional relationship between depression and personality pathology over the course of treatment. It is important to note, however, that all of these prior studies relied upon the outdated categorical approach for PD classification, which has been criticized regarding its lack of clinical utility, reliability, and validity (Hengartner, Zimmermann, & Wright, 2018).

Studies employing dimensional measures of personality have traditionally largely focused on the Big Five traits, and indeed provided some evidence for associations with treatment response (Bucher, Suzuki, & Samuel, 2019). In depressed patients, lower scores on emotional stability, extraversion, and openness have been observed to be predictive of poorer treatment outcomes (Quilty et al., 2008). The Big Five also appear to be changeable following clinical interventions and a recent meta-analysis found that particularly emotional stability/neuroticism and extraversion increased following treatment (Roberts et al., 2017). These personality traits are most strongly linked to individuals' affect, whereby neuroticism is associated with negative and extraversion with positive affect (McNiel & Fleeson, 2006). As a consequence, these traits may constitute ideal intervention targets as psychotherapeutic techniques aim to increase positive (e.g. fostering rewarding social interactions) and decrease negative affect (e.g. fostering strategies to cope with self-criticism). Overall, this research indicates the potential benefit of assessing personality traits dimensionally in clinical psychiatric contexts. The Big Five depict common personality features, however, and were not constructed to capture maladaptive variants of personality such as the dimensional classification approaches incorporated in the DSM-5 and ICD-11 (APA, 2013; Tyrer, Mulder, Kim, & Crawford, 2019). For example, in the Alternative Model for Personality Disorders (AMPD) in DSM-5 Section III (Krueger, Derringer, Markon, Watson, & Skodol, 2012), PDs are classified dimensionally in terms of impairments in personality functioning (criterion A) and maladaptive personality traits (criterion B). Criterion B comprises a hierarchical model including five maladaptive trait domains (negative affectivity, detachment, antagonism, disinhibition, psychoticism) on a higher level and 25 trait facets at a subordinate level. Four of the five maladaptive trait domains are substantially associated with the Big Five and can therefore be considered as maladaptive variants of the Big Five (i.e. negative affectivity is negatively associated with emotional stability, detachment with extraversion, agreeableness with antagonism, and conscientiousness with disinhibition; Suzuki, Griffin, & Samuel, 2017). To date, it remains unclear whether maladaptive personality traits are changeable following treatment and if they can predict treatment response in depression.

Psychotherapeutic treatments for depression differ in the extent that they were meant to target personality pathology. The recommended first-line treatment for depression (Middleton, Shaw, Hull, & Feder, 2005) is CBT (Beck, 1976; Beck, Rush, Shaw, & Emery, 1979), which primarily aims to improve MDD severity by altering depressive cognitive appraisals (e.g. ‘I am worthless and the future is hopeless’) and dysfunctional behaviours such as social withdrawal and physical inactivity (Garratt, Ingram, Rand, & Sawalani, 2007). In contrast, schema therapy (ST) was originally developed for the treatment of CBT non-respondents including patients with PDs (Young, 1990) and targets so-called early maladaptive schemas (EMSs; Young, Klosko, & Weishaar, 2003) such as ‘Emotional deprivation’ or ‘Failure’. In ST, EMSs are considered as dysfunctional trait-like patterns of thoughts, memories, emotions, physical sensations, and attention tendencies, which are relatively enduring and repeatedly re-activated throughout life. They are believed to emerge during early childhood and particularly in adverse social environments including emotional neglect or physical abuse, in which the child's basic psychological needs such as attachment or autonomy are unmet. Recently, a large overlap between ST concepts and maladaptive personality traits has been reported (Bach & Bernstein, 2019; Bach, Lee, Mortensen, & Simonsen, 2016), which may indicate that ST is particularly well equipped for the treatment of maladaptive trait domains. Specifically, although CBT tries to challenge dysfunctional cognitions, for example using Socratic dialogue, and to reduce dysfunctional behaviours through behavioural activation (Sudak, 2012), ST applies more experiential and emotion-focused strategies such as imagery rescripting, mode dialogues on chairs, and ‘limited reparenting’ techniques (Young et al., 2003). In an initial RCT, ST was found to be similarly effective in the treatment of depression compared to CBT (Carter et al., 2013). ST was also found to be effective in the treatment of PDs (Bamelis, Evers, Spinhoven, & Arntz, 2014). To date, however, no study has been conducted investigating whether ST is better suited than CBT for the treatment of maladaptive personality traits in depressed patients.

Conversely, it is also unclear if ST or CBT are more effective in the treatment of depression in individuals with certain maladaptive personality traits. Addressing these questions could help to come closer to answering the old but pressing question ‘What works best for whom’ (Paul, 1967).

### The present study

This study used data from an RCT comparing CBT and ST in patients with depression in psychiatric and day clinical settings (Kopf-Beck et al., 2020). Based on the mixed meta-analytic findings of Newton-Howes et al. (2014) and Banyard et al. (2021), we first explored whether higher pre-treatment levels of maladaptive trait domains were associated with smaller changes in depression symptom severity from pre- to post-treatment, and/or *vice versa*. Second, we investigated the changeability of the five maladaptive trait domains over the course of treatment and hypothesized their reduction particularly for negative affectivity and detachment in line with previous findings on corresponding Big Five traits (Roberts et al., 2017). Third, we tested differential effectiveness of CBT and ST in targeting maladaptive trait domains and hypothesized that ST would outperform CBT as it was specifically developed for the treatment of PDs. Finally, we examined whether the maladaptive trait domains moderate differential treatment effects of ST and CBT in depression.

## Methods

### Sample

We used data from the OPTIMA study (OPtimized Treatment Identification at the MAx Planck Institute of Psychiatry; Kopf-Beck et al., 2020), an RCT investigating the effectiveness of CBT, ST, and individual supportive therapy (IST) in depressed patients admitted to a psychiatric hospital for inpatient or day clinic treatment. Inclusion criteria comprised a primary diagnosis of depression with at least moderate severity. This was rated as ⩾20 according to Beck Depression Inventory-II (BDI-II; Hautzinger, Keller, & Kühner, 2009) *or* the Montgomery–Åsberg Depression Rating Scale (Montgomery & Åsberg, 1979). Additional requirements were proficiency in the German language and age between 18 and 75 years. Main exclusion criteria were lifetime diagnosis of bipolar disorder or schizophrenia, acute psychotic disorder, acute suicidality, concomitant substance use disorder, or organic mental disorders (for more details see Kopf-Beck et al., 2020). Clinical diagnoses of depression and comorbid mental disorders were assessed by trained raters using the Composite International Diagnostic Interview (CIDI). This study was conducted in accordance with Declaration of Helsinki Standards and all patients provided written informed consent prior to participation.

In total, *N* = 300 depressed patients fulfilled inclusion criteria and were randomized in a parallel group design to CBT, ST, or IST. For the purpose of this study, we selected participants randomized to the ST and CBT treatment arm (*N* = 199). We focused on ST and CBT because these conditions enabled us to perform a theory-driven test as to whether a psychotherapy developed for the treatment of non-responsive patients with PD characteristics (i.e. ST) was superior to the gold-standard psychotherapy for depression (i.e. CBT) in reducing maladaptive personality traits. However, we repeated the analyses regarding the first and second research questions using data from the full sample.

Altogether six participants were excluded since they (i) did not reach the defined cut-off score for depression (*n* = 2), (ii) dropped out before receiving the first psychotherapy session (*n* = 1), (iii) failed to complete baseline questionnaires before commencing treatment (*n* = 2), or (iv) fulfilled an exclusion criterion that was identified retrospectively (*n* = 1). This resulted in a final sample of *N* = 193 participants (53.4% women, *M*_age_ = 42.9, s.d. = 13.4). Patients reported an average BDI-II pre-treatment score of 31.4 (s.d. = 8.7) indicating severe depressive symptoms. Average pre-treatment mean scores of PID-5-FBF domain scales ranged from ‘normal’ to clinically elevated ‘mild’ levels with 0.55 (T53) for psychoticism and 1.21 (T61) for detachment, respectively (Rek, Kerber, Kemper, & Zimmermann, 2021). However, there was also substantial heterogeneity between patients as indicated in ranges from normal to clinically relevant levels (online Supplementary Fig. S1). About 28% of patients who completed the Assessment of DSM-IV Personality Disorders (ADP-IV; Doering et al., 2007) qualified for a comorbid PD (see online Supplementary Table S1). At pre-treatment, patients of both treatment arms did not differ with regards to socio-demographic characteristics and psychiatric symptom severity (see Table 1).

**Table 1.** Demographic and clinical variables at baseline (*N* = 193)

Characteristics	CBT (*N* = 100) mean (s.d.) or *n* (%)	ST (*N* = 93) mean (s.d.) or *n* (%)	*p* Value
Sex (women)	50 (50.0)	53 (57.0)	0.408
Age (years)	42.18 (14.11)	43.63 (12.61)	0.452
German nationality	93 (92.5)	83 (11.6)	0.497
Other nationality			0.260
Afghani	0 (0.0)	1 (1.1)	
Austrian	0 (0.0)	2 (2.2)	
Brazilian	0 (0.0)	1 (1.1)	
Croatian	2 (2.0)	0 (0.0)	
Estonian	1 (1.0)	0 (0.0)	
French	1 (1.0)	1 (1.1)	
Hungarian	1 (1.0)	0 (0.0)	
Persian	0 (0.0)	1 (1.1)	
Polish	0 (0.0)	2 (2.2)	
Russian	0 (0.0)	1 (1.1)	
Turkish	2 (2.0)	2 (2.2)	
US American	1 (1.0)		
Employment			0.605
Employed	56 (56.0)	46 (49.5)	
Unemployed	36 (36.0)	40 (43.0)	
Not indicated	8 (8.0)	7 (7.5)	
Monthly income in €			0.842
<500	13 (13.0)	6 (6.5)	
500–1000	7 (7.0)	9 (9.7)	
1000–1500	11 (11.0)	9 (9.7)	
1500–2000	7 (7.0)	8 (8.6)	
2000–3000	19 (19.0)	20 (21.5)	
3000–4000	12 (12.0)	14 (15.1)	
4000–5000	8 (8.0)	10 (10.8)	
>5000	14 (14.0)	10 (10.8)	
Not indicated	9 (9.0)	7 (7.5)	
Marital status			0.388
Divorced	9 (9.0)	3 (3.2)	
Engaged	2 (2.0)	0 (0.0)	
In a relationship	17 (17.0)	21 (22.6)	
Separated	5 (5.0)	1 (1.1)	
Married	33 (33.0)	31 (33.3)	
Other	1 (1.0)	2 (2.2)	
Single	25 (25.0)	26 (28.0)	
Widowed	1 (1.0)	2 (2.2)	
Not indicated	7 (7.0)	7 (7.5)	
Education			0.556
A-Level	53 (53.0)	44 (47.3)	
High school	21 (21.0)	23 (24.7)	
None	3 (3.0)	1 (1.1)	
Other	11 (11.0)	8 (8.6)	
Secondary school	12 (12.0)	17 (18.3)	
Baseline scores			
BDI-II: Depression	30.79 (8.48)	32.14 (8.99)	0.284
PID-5-FBF: Neg. affectivity	1.37 (0.39)	1.48 (0.39)	0.050
PID-5-FBF: Detachment	1.12 (0.51)	1.13 (0.55)	0.835
PID-5-FBF: Antagonism	0.56 (0.33)	0.66 (0.43)	0.068
PID-5-FBF: Disinhibition	1.08 (0.37)	1.15 (0.41)	0.175
PID-5-FBF: Psychoticism	0.52 (0.45)	0.58 (0.48)	0.370
CIDI: Any comorbidity			
Psychoactive substance abuse	44 (44.0)	38 (40.9)	0.768
Anxiety disorder	81 (81.0)	72 (77.4)	0.663
Somatoform disorder	44 (44.0)	43 (46.2)	0.867
Eating disorder	2 (2.0)	7 (7.5)	0.139

CBT, cognitive behavioural therapy; ST, schema therapy; CIDI, Composite International Diagnostic Interview; s.d., standard deviation.

*p < 0.01; **p < 0.001.

### Intervention

After providing informed consent and completing baseline assessment (see Kopf-Beck et al., 2020 for details), patients received a course of psychotherapy for 7 weeks. In addition to psychotherapy, patients received inpatient/day-clinic treatment as usual including pharmaco-, exercise-, socio-, and/or occupational-therapy (see online Supplementary material for additional information on concomitant treatments). During the intervention phase, patients received two group (100 min each) and two individual (50 min each) manual-based therapy sessions per week and were excluded if they missed more than six out of a maximum of 28 psychotherapy sessions. To ensure adherence with the study manuals and quality, trial therapists were trained by leading experts in CBT and ST and received monthly supervision. Therapy sessions were videotaped and a randomly selected subsample rated by two independent raters indicated high to very adherence to the manual. Regarding concomitant pharmacotherapy regimens, there were no significant differences between treatment arms (see online Supplementary Table S2).

The interventions are described in more detail in Kopf-Beck et al. (2020). Briefly, CBT was based on a modified and extended version of an established treatment manual for MDD by Hautzinger (2013). The manual consists of five modules including (a) psychoeducation, (b) behavioural activation, (c) modification of dysfunctional attitudes and automatic thoughts, (d) social competence training, and (f) relapse prevention. ST was delivered according to the ST manual designed for a psychiatric inpatient and day clinic setting (Egli et al., 2019). The manual contains three treatment phases with the main focus on (a) psychoeducation, (b) modification of EMS and dysfunctional modes, and, (c) exercises on strategies for transfer and relapse prevention.

### Measures

PID-5-FBF and BDI-II were assessed at pre- and post-treatment for main analyses. The self-report version of the ADP-IV was used for additional description of the sample (see details in online Supplementary material). Note that the ADP-IV was added to the study later on, so was only available for a subset of patients.

#### Beck Depression Inventory II

We used the German version of the BDI-II (Beck, Steer, & Brown, 1996; German version: Hautzinger et al., 2009) to assess the severity of depressive symptoms. The self-report scale entails 21 items, which are rated on a 4-point Likert scale ranging from 0 to 3. Higher scores indicate greater depressive symptom levels. Psychometric properties of the scale have been reported in previous research (Kühner, Bürger, Keller, & Hautzinger, 2007). For our analyses, we computed three BDI parcels by splitting the items into three groups of 7 items each and computing the mean score. See online Supplementary materials for details on psychometric properties.

#### Personality inventory for DSM-5 faceted brief form (PID-5-FBF)

The German version (Zimmermann et al., 2014) of the 100-item (Maples et al., 2015) self-report questionnaire PID-5 (Krueger et al., 2012) entails 25 subscales representing the trait facets of AMPD's trait model. The three most important facets were used to define each of the five maladaptive trait domains (i.e. negative affectivity, detachment, antagonism, disinhibition, and psychoticism). The items are rated on a 4-point Likert scale ranging from 0 = *very false* to 3 = *very true*, with higher scores indicating greater personality pathology. See online Supplementary materials for details on psychometric properties.

### Statistical analyses

All analyses were conducted using statistical software R (R Core Team, 2020) and the *psych* (Revelle, 2020), *lavaan* (Rosseel et al., 2018), and *EffectLiteR* packages (Mayer, Dietzfelbinger, Rosseel, & Steyer, 2016). Scripts for analyses are available in the online Supplementary material.

**Fig. 1.**
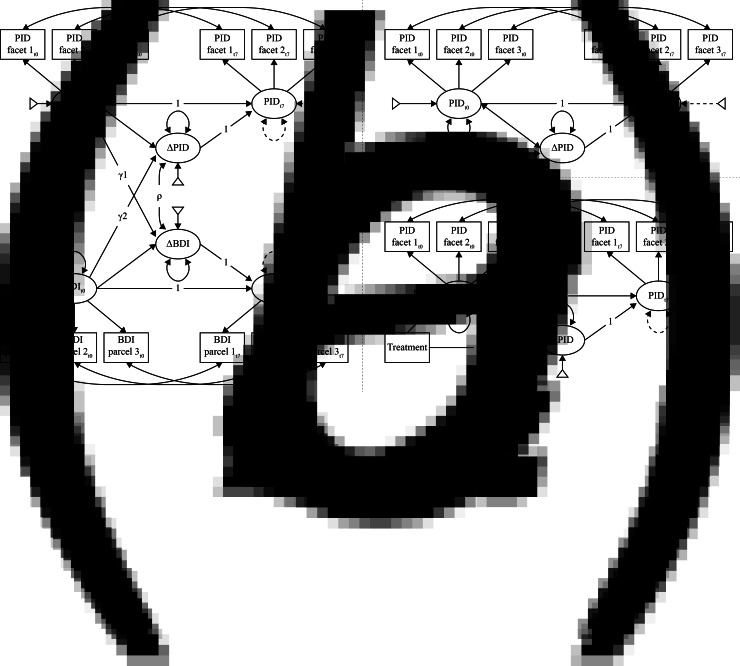
LCS models to address research questions 1–3. Dashed lines show effects and variances fixed to zero and ‘1's indicate effects fixed to 1. Lines with two arrowheads represent (co-)variances while lines with one arrowhead show regression effects. Circles indicate latent variables, rectangles display manifest variables, and triangles show intercepts. Intercepts and residual variances of manifest variables are omitted. All LCS models depicted in this figure are based on scalar measurement invariance, whereby loadings and intercepts of manifest variables (i.e. parcels and core facets) are fixed to be equal, but residual variances are allowed to be different across time. (*a*) Bivariate LCS model, which uses regressions of change scores on baseline levels to assess the extent to which changes in depressive symptoms between pre- (T1) and post-treatment (T2) are a function of baseline maladaptive traits (*γ*1), *vice versa* (*γ*2) or both. In the bivariate LCS model, associations between depressive symptoms and maladaptive traits at pre-treatment (*Φ*) as well as between depressive symptom and maladaptive trait change scores (*ρ*) were also explored. (*b*) (Unconditional) univariate LCS model, in which the effect of interest was the intercept of change (Δ*μ*) and the association of baseline maladaptive traits and maladaptive trait change score was modelled as a covariance. (c) Analyses of the treatment covariate. In this model, the association of baseline maladaptive traits and maladaptive trait change scores was modelled as a self-feedback effect as in bivariate LCS models.

Throughout the first three research questions, we used latent change score (LCS; McArdle, 2009) models with multiple indicators and scalar measurement invariance across time for each construct (see Fig. 1). Maladaptive trait domains were defined by the three most relevant PID-5-FBF facet scores, and depressive symptoms were defined by three parcels each consisting of seven BDI items. To address the first research question, we used five bivariate LCS models (panel a), each time including depressive symptoms and one of the five maladaptive trait domains. The focal parameters *γ*1 and *γ*2 (i.e. ‘cross-domain coupling’; Kievit et al., 2018) represent the extent to which changes in construct *X* (e.g. depressive symptoms) between pre- and post-treatment are a function of construct *Y* (e.g. detachment) at pre-treatment, while controlling for pre-treatment values of construct *X*,or *vice versa*. To test our second research question on the changeability of the maladaptive trait domains from pre- to post-treatment, five univariate LCS models (panel b) were estimated. Particularly, we tested the (unconditional) intercept of change in the maladaptive trait domain (Δ*μ*_domain_) for significance. Third, treatment effects (*β*) of ST *v.* CBT on changes in the five maladaptive trait domains were tested by including treatment as predictor in univariate LCS models (panel c).

Finally, to explore whether the treatment effects are moderated by the five maladaptive trait domains, we applied multi-group structural equation modelling using EffectLiteR (Mayer et al., 2016, 2020). This approach not only allows for testing whether there is an average effect of CBT compared to ST with regards to depressive symptoms, but it also estimates whether treatment effects are conditional on pre-treatment maladaptive trait domains. In particular, we estimated a structural equation model with two groups (CBT and ST), using post-treatment depressive symptoms as the latent outcome variable, pre-treatment depressive symptoms as a latent covariate and the five maladaptive trait domains as observed covariates. EffectLiteR provides an omnibus test for the null hypothesis that there are no interactions between treatment condition and covariates (i.e. that effects of covariates do not differ between groups).

Across analyses, missing values in structural equation models were handled using a full information maximum likelihood approach (Enders & Bandalos, 2001). To account for multiple comparisons, a conservative alpha error rate of 0.01 was selected, which equals Bonferroni correction with alpha error rate of 0.05 for statistical tests across five maladaptive personality traits.

## Results

Table 2 summarizes the focal standardized parameter estimates from the LCS models (see online Supplementary Table S3 for fit indices). Maladaptive traits and depressive symptoms did not show cross-domain coupling using bivariate LCS models (i.e. *γ*1 and *γ*2 were non-significant). Accordingly, pre-treatment levels of the respective maladaptive trait domains were not associated with rates of change in depressive symptom severity over the course of treatment, or *vice versa*. Yet, with the exception of antagonism, we observed substantial correlations of maladaptive trait domains with depressive symptoms at pre-treatment (standardized *Φ* ranged from 0.37 to 0.52) and residuals of their respective change scores (standardized *ρ* ranged from 0.32 to 0.70). Repeating the analyses including the IST condition showed the same pattern of results, although there was evidence for a small cross-coupling effect of baseline detachment on changes in depressive symptoms (online Supplementary Table S4).

**Table 2.** Standardized parameters of LCS models

Model	Parameter	PID-5-FBF				
		Negative affectivity	Detachment	Antagonism	Disinhibition	Psychoticism
Bivariate LCS models	*Φ*	0.368*	0.521**	0.177	0.460*	0.386**
	*γ*1	0.065	0.241	−0.006	0.015	0.153
	*γ*2	0.024	0.112	−0.094	−0.163	0.023
	*ρ*	0.503**	0.696**	0.096	0.482**	0.316
Univariate LCS models	Δ*μ*	−0.724**	−0.736**	−0.376*	−0.890**	−0.522**
Univariate LCS models including treatment	*β*	0.054	0.102	0.186	0.280	0.061

LCS, latent change score; PID-5-FBF, Personality Inventory for DSM-5 Faceted Brief Form.

*Note.* Analyses are based on the final sample of *n* = 193. *Φ* = depression and maladaptive trait domain correlation at baseline; *γ*1 = cross-domain coupling: trait domain score at baseline predicting rate of change in depression; *γ*2 = cross-domain coupling: depression score at baseline predicting rate of change in trait domain; *ρ* = correlated residuals of depression and maladaptive trait domain change scores; Δ*μ* = intercept of maladaptive trait domain change score; *β* = effect of treatment group on change scores of maladaptive trait domains.

*p < 0.01; **p < 0.001.

**Fig. 2.**
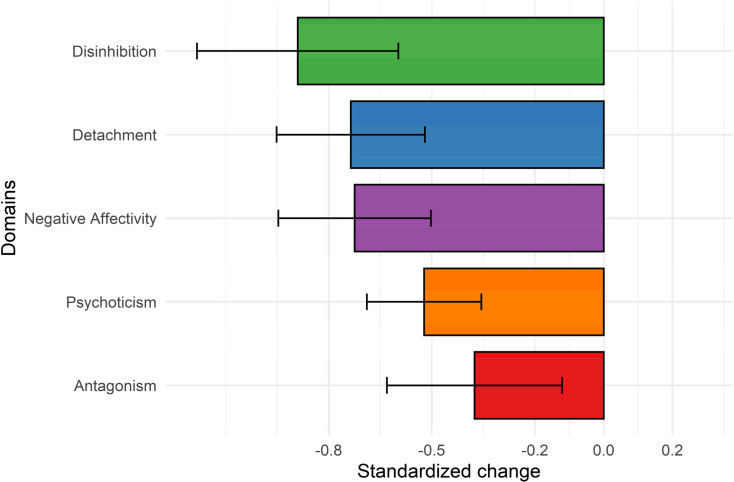
Standardized mean changes in maladaptive traits following treatment as estimated using univariate LCS models (see Table 2). Error bars represent 95% confidence intervals for standardized mean change scores. See online Supplementary Figure S3 for standardized mean changes broken down to individual maladaptive personality facet scores.

Applying univariate LCS models, negative change score intercepts for each of the maladaptive traits were observed after 7 weeks of psychiatric treatment for depression (standardized Δ*μ*_domain_ ranged from −0.38 to −0.89; see Fig. 2). Results from these analyses – and analyses including the IST condition (see online Supplementary Table S4) – indicated that maladaptive traits can change during clinical interventions, particularly negative affectivity, detachment, and disinhibition. Analysing individual facets to explore results further indicated largest changes for depressivity, anxiousness, anhedonia, distractibility, and withdrawal (online Supplementary Fig. S3). When adding the treatment condition (CBT *v.* ST) as an additional predictor to the univariate LCS models, both treatment types were comparably effective in the reduction of the five maladaptive traits (i.e. *β* was non-significant).

Results of the multi-group structural equation modelling based on EffectLiteR indicated that the average treatment effect on post-treatment depressive symptoms was not significant (Wald χ^2^ = 2.80, df = 1, *p* = 0.09; see online Supplementary Table S5 for fit indices and online Supplementary Table S6 for regression coefficients), suggesting a comparable effectiveness of CBT and ST in the treatment of depression. In addition, we found no evidence for conditional treatment effects indicated by covariate-treatment interactions (Wald χ^2^ = 6.38, df = 6, *p* = 0.38).

## Discussion

Due to the high rates of relapse and non-response in depression, it is key to identify factors that may obstruct treatment responsiveness and could inform treatment selection for the individual patient (Cohen & DeRubeis, 2018; Fournier et al., 2009). Using RCT data, we investigated the role of personality pathology over the course of 7-week inpatient or day clinic depression treatments by studying the impact and changeability of maladaptive personality traits according to the recently developed AMPD. Our results indicated that maladaptive traits were not predictive of changes in depressive symptomatology at post-treatment, or *vice versa*. Moreover, while we observed overall reductions in maladaptive traits at the end of treatment, changes were similar in magnitude in CBT and ST arms consistent with the null hypothesis of an absence of differential treatment effectiveness. Finally, inter-individual differences in maladaptive traits prior to treatment could not inform favourable allocation to CBT *v.* ST in the treatment of depression. While these findings did not provide suggestions for new treatment targets or allocation choices, they again disconfirm the old clinical myths that personality pathology is stable over time, not amenable to change, and worsens treatment outcomes of depressed patients.

Previous meta-analyses reported that personality pathology negatively impacts psychotherapeutic treatment outcomes of depressed patients (Banyard et al., 2021; Newton-Howes et al., 2014). Notably, Banyard et al. (2021) conducted several sensitivity analyses in subgroups of high-quality trials and adjusting for baseline depression severity to assess the robustness of effects. Results of these sensitivity analyses are in line with our results showing no detrimental effects of the five maladaptive trait domains on treatment outcome. Our findings support a co-occurrence of personality pathology and depression levels at pre-treatment and co-occurrence of their respective change scores during treatment. Baseline co-occurrence of the two constructs in our study suggests that depressed patients with greater levels of personality pathology may be more severely depressed at pre-treatment. This has also been reported by Banyard et al. (2021), who stated that ‘[t]he apparent effect of PD on depression outcomes is likely explained by higher intake severity rather than treatment resistance’. Regarding the associated change scores of maladaptive trait domains and depression, more research is needed to investigate the observed correlations due to challenges of their interpretation in intervention studies (Könen & Karbach, 2021). As such, putative explanations may be a shared underlying process/transdiagnostic factor such as emotion dysregulation targeted during treatment (Abdi & Pak, 2019) or a third variable such as therapeutic alliance that affects both changes (Horvath, Del Re, Flückiger, & Symonds, 2011). However, results could also indicate that reciprocal, more dynamic processes of changes in maladaptive trait domains and depression levels occurred, which we were not able to capture with two time points only. Thus, future research should investigate the potential underlying variables/processes, while using more frequent assessment time points to capture potential dynamic processes of change (Renner et al., 2018).

PDs have originally been conceptualized and defined as ‘an enduring pattern of inner experience and behavior that […] is pervasive and inflexible, has an onset in adolescence or early adulthood, is stable over time, and leads to distress or impairment’ (APA, 2013, p. 685). The stability of personality pathology has been supported by Wright et al.'s (2015) observational study on maladaptive trait domains’ mean-level change and rank-order stability over a time period of 1.4 years. Additionally, Zimmermann et al. (2017) found that individual differences in maladaptive traits were relatively stable and rarely affected by situational factors. In the context of these findings, we observed substantial decreases in maladaptive traits after a relatively time-limited psychiatric inpatient treatment period of 7 weeks, which highlights the potential of clinical interventions to enable changes in personality pathology. Here, besides disinhibition among the largest reductions were observed for negative affectivity and detachment, which aligns with Roberts et al.'s (2017) meta-analytic findings of general adaptive changes in Big Five traits that included particularly pronounced increases in emotional stability (opposite pole of negative affectivity) and extraversion (opposite pole of detachment) over the course of treatment. Of note, emotional stability has been associated with lower levels of negative affect and extraversion with greater positive affect (McNiel & Fleeson, 2006) and clinical interventions for depression specifically target these traits. In sum, we show evidence in favour of the malleability of personality pathology during clinical interventions at least in the short run, which may reassure clinical practitioners. However, investigation of long-term effectiveness of such short clinical interventions for personality pathology is warranted (e.g. by using follow-up data of at least 6 months).

Contrary to our expectations, we observed no differences in average effects of ST and CBT in the treatment of maladaptive trait domains and no benefit of using these traits to inform personalized treatment choice to ST or CBT. These results could be explained by an overall relatively low treatment dosage and short treatment duration of 7 weeks for ST, for which effectiveness has been observed to increase with the overall number of sessions and time of treatment (Jacob & Arntz, 2013). Alternatively, identification of specific psychotherapeutic treatment effects may have been obfuscated by the psychiatric inpatient setting, in which patients continued to receive standard care (e.g. pharmaco- or exercise therapy) during the course of treatment. These concomitant interventions have been shown to be effective treatments on their own (Fournier et al., 2010; Kvam, Kleppe, Nordhus, & Hovland, 2016), so replication in an outpatient setting and/or including a separate treatment arm for pharmacotherapy is warranted. However, our findings also align well with numerous studies showing equal effectiveness of different psychotherapies in line with the ‘Dodo Bird Verdict’ (Wampold, 2015). That is, factors that are common to different therapeutic approaches such as therapeutic alliance or empathy are assumed to explain most of the variance in treatment outcome (30–70%; Imel & Wampold, 2008). Finally, different forms of psychotherapies may indeed be comparable in their effectiveness but this effectiveness could be mediated by different mechanisms (DeRubeis, Brotman, & Gibbons, 2005). Thus, more research is needed to advance our understanding on the optimal treatment duration of ST for personality pathology, which is currently underway (Kool et al., 2018), and on investigating mechanisms underlying different psychotherapeutic interventions.

## Limitations

Our study needs to be interpreted in light of its limitations. First and foremost, the assessment of maladaptive trait domains and depression was only based on self-reports. Compared to other forms of psychopathology, PDs are considered to be relatively ego-syntonic meaning that affected individuals often present with poor insight or little awareness into their dysfunctional patterns of thoughts and behaviours. As such, self-reports such as the PID-5-FBF may be confounded by distorted representations of self and others (i.e. impairments of identity and empathy; APA, 2013) or alternatively, by a negative affect bias of depressed patients. Thus, future studies may benefit from inclusion of clinical expert ratings (Morey, Krueger, & Skodol, 2013) or informant reports (Markon, Quilty, Bagby, & Krueger, 2013). Second, we only focused on assessment of criterion B (i.e. maladaptive trait domains). Although studies have highlighted a great overlap between criteria A and B (Zimmermann et al., 2015), assessment of criterion A could have helped to determine more general impairments in personality functioning, which are supposed to underlie all types of PDs (Morey et al., 2011). Third, our sample size may have been too small to detect differences between CBT and ST or to inform personalized treatment allocation choices, so even larger sample sizes with at least 300 participants per arm are needed in future research (Luedtke, Sadikova, & Kessler, 2019). Fourth, treatment effectiveness was evaluated based on BDI-II and PID-5-FBF scores at the end of treatment. Yet, previous research has highlighted the importance of including other outcomes (e.g. social functioning) to assess the potential impact of personality pathology and follow-up assessments (e.g. at 6-month post-intervention) to measure more enduring treatment effects (Newton-Howes, Mulder, Ellis, Boden, & Joyce, 2018), which should be addressed in future trials. Fifth, we were unable to control our analyses for medications and concomitant treatments, which could have influenced results from LCS models. Finally, it may be valuable to include a wait-list control arm in future studies to test if changes in personality pathology were not simply due to regression to the mean but rather a consequence of treatment.

## Conclusion

Our study contributes to a better understanding of the effects of personality pathology on treatment outcomes of depressed patients. Contrary to clinical experiences, personality pathology did not negatively impact treatment outcome (i.e. depressive symptomatology) of depressed patients or *vice versa*. Additionally, maladaptive traits showed a substantial decrease after an intensive, yet relatively brief clinical intervention, which was irrespective of the psychotherapeutic treatment type. We also observed co-occurrences between depression and maladaptive trait domains at pre-treatment and between their respective changes over the course of treatment, which suggests that either a third variable affects both changes or reciprocal, or more dynamic processes of changes in maladaptive trait domains and depression occurred. As such, determination of the mechanisms linking the development of both personality pathology and depressive symptoms, as well as their changes will likely shed further light on the underlying causes, which could open up the possibility of new prevention and treatment targets.

## Supplementary material

For supplementary material accompanying this paper visit https://doi.org/10.1017/S0033291722001209. click here to view supplementary material
