# Supplementary material for: Evaluating the role of maladaptive personality traits in schema therapy and cognitive behavioural therapy for depression
Source: Psychol Med. 2022 May 10;53(10):4405–14. doi: 10.1017/S0033291722001209 (PMC10388330; doi:10.1017/S0033291722001209)
Supplement: Supplementary file 1 [file S0033291722001209sup001.docx]

**Supplementary Material**

**Measures**

**Beck Depression Inventory II (BDI-II)**. BDI parcels were constructed using (i) items 1, 4, 7, 10, 13, 16, 19 for parcel 1, (ii) items 2, 5, 8, 11, 14, 17, 20 for parcel 2, and (iii) items 3, 6, 9, 12, 15, 18, and 21 for parcel 3. Internal consistencies for the BDI parcels were poor to questionable at pre-treatment (Cronbach’s α range: .57 to .66) and acceptable to good at post-treatment (Cronbach’s α range: .74 to .81). Pre- to post-treatment correlations of parcels were moderate (Pearson’s *r* range: .42-.48).

**Personality Inventory for DSM-5 Faceted Brief Form (****PID-5-FBF).** The psychometric properties of the PID-5 scales have been reported to be sound (Zimmermann et al., 2019) with test-retest correlations between .70 and .87 for the five domains using the PID-5 SF (Díaz-Batanero et al., 2019). In our sample, internal consistencies of the PID-5-FBF facets were good (Cronbach’s α ranging from .84 to .88 at pre-treatment and .81 to .89 at post-treatment) and pre- to post-treatment correlations of facet scores were large (Pearson’s *r* range: .67-.80).

**Assessment of DSM-IV Personality Disorders (ADP-IV).** The Assessment of DSM-IV Personality Disorders (ADP-IV; Doering et al., 2007) is a 94 item self-report questionnaire assessing the criteria of the 12 PDs of DSM-IV categorically and dimensionally. First, each item has to be answered on a 7-point Likert scales and second, for ratings >4, additional distress has to be assessed on a 3-point Likert scale. We used the conservative, categorical scoring of the ADP-IV to compare results for maladaptive personality traits to classical diagnostic categories. To qualify for a categorical diagnosis, patients had to exhibit >3 or >4 items (depending on diagnostic category) with Likert scale severity >4 without considering the distress scale. Psychometric properties were demonstrated to be sound (Schotte et al., 2004).

**Concomitant care**

The psychotherapy program was embedded in a comprehensive inpatient-/day clinic treatment protocol. Beside pharmacotherapy, major components of the additional treatment were occupational- and exercise-therapy, case management, and relaxation- and cognitive training accounting for 94.7% of all additional co-therapy sessions. None of these components overlapped with the treatment interventions of ST or CBT, even though, we expect the concomitant care to act in an anti-depressant manner overall. Overall, psychotherapy was the major treatment component accounting for 39.2% of all delivered interventions apart from pharmacotherapy.

**Table S1**

*Additional personality characteristics of the final sample*

| Assessment of DSM-IV Personality Disorders (ADP-IV) | CBT (n=100) | ST (n=93) | p |
| --- | --- | --- | --- |
| Antisocial (%) |  |  | .545 |
| Yes | 0 (0.0) | 1 (1.1) |  |
| No | 66 (66.0) | 63 (67.7) |  |
| Not assessed | 34 (34.0) | 29 (31.2) |  |
| Borderline (%) |  |  | .042 |
| Yes | 2 (2.0) | 10 (10.8) |  |
| No | 64 (64.0) | 54 (58.1) |  |
| Not assessed | 34 (34.0) | 29 (31.2) |  |
| Depressive (%) |  |  | .524 |
| Yes | 5 (5.0) | 3 (3.2) |  |
| No | 58 (58.0) | 61 (65.6) |  |
| Not assessed | 37 (37.0) | 29 (31.2) |  |
| Dependent (%) |  |  | .858 |
| Yes | 2 (2.0) | 3 (3.2) |  |
| No | 63 (63.0) | 57 (61.3) |  |
| Not assessed | 35 (35.0) | 33 (35.5) |  |
| Histrionic (%) |  |  | .321 |
| Yes | 0 (0.0) | 2 (2.2) |  |
| No | 66 (66.0) | 62 (66.7) |  |
| Not assessed | 34 (34.0) | 29 (31.2) |  |
| Narcissistic (%) |  |  | .545 |
| Yes | 0 (0.0) | 1 (1.1) |  |
| No | 66 (66.0) | 63 (67.7) |  |
| Not assessed | 34 (34.0) | 29 (31.2) |  |
| Passive-aggressive (%) |  |  | .57 |
| Yes | 0 (0.0) | 1 (1.1) |  |
| No | 66 (66.0) | 62 (66.7) |  |
| Not assessed | 34 (34.0) | 30 (32.3) |  |
| Paranoid (%) |  |  | .074 |
| Yes | 1 (1.0) | 7 (7.5) |  |
| No | 64 (64.0) | 57 (61.3) |  |
| Not assessed | 35 (35.0) | 29 (31.2) |  |
| Schizotypal (%) |  |  | .321 |
| Yes | 0 (0.0) | 2 (2.2) |  |
| No | 66 (66.0) | 62 (66.7) |  |
| Not assessed | 34 (34.0) | 29 (31.2) |  |
| Schizoid (%) |  |  | .812 |
| Yes | 2 (2.0) | 3 (3.2) |  |
| No | 64 (64.0) | 61 (65.6) |  |
| Not assessed | 34 (34.0) | 29 (31.2) |  |
| Avoidant (%) |  |  | .915 |
| Yes | 7 (7.0) | 7 (7.5) |  |
| No | 59 (59.0) | 57 (61.3) |  |
| Not assessed | 34 (34.0) | 29 (31.2) |  |
| Obsessive-compulsive (%) |  |  | .891 |
| Yes | 4 (4.0) | 5 (5.4) |  |
| No | 60 (60.0) | 54 (58.1) |  |
| Not assessed | 36 (36.0) | 34 (36.6) |  |
| Any personality disorder (%) |  |  | .304 |
| Yes | 13 (13.0) | 19 (20.4) |  |
| No | 47 (47.0) | 36 (38.7) |  |
| Not assessed | 40 (40.0) | 38 (40.9) |  |

**Table S2**

*Dose of medication and change in medication at baseline and over the course of treatment between CBT and ST.*

|  |  | ST  (*n* = 95) | CBT  (*n* = 101) | Chi-squared, df, p-value  (Kruskal-Wallis) |
| --- | --- | --- | --- | --- |
| Medication at baseline ^a^ | | | | |
|  | Antidepressants ^b^ | 81 | 85 | *X^2^* = 0.046, *df* = 1, *p* = .830 |
|  | Mood stabilizer | 19 | 11 | *X^2^* = 3.117, *df* = 1, *p* = .077 |
|  | Neuroleptics | 24 | 26 | *X^2^* = 0.006, *df* = 1, *p* = .939 |
|  | Tranquilizer | 12 | 18 | *X^2^* = 1.012, *df* = 1, *p* = .314 |
|  | **TOTAL** | **83** | **88** | *X^2^* = 0.003, *df* = 1, *p* = .960 |
| Medication over course of treatment ^a^ | | | | |
|  | Antidepressants ^b^ | 87 | 93 | *X^2^* = 0.016, *df* = 1, *p* = .899 |
|  | Mood stabilizer | 24 | 17 | *X^2^* = 2.093, *df* = 1, *p* = .148 |
|  | Neuroleptics | 35 | 37 | *X^2^* = 0.001, *df* = 1, *p* = .976 |
|  | Tranquilizer | 25 | 21 | *X^2^* = 0.827, *df* = 1, *p* = .363 |
|  | **TOTAL** | **88** | **94** | *X^2^* = 0.014, *df* = 1, *p* = .906 |
| Number of changes in medication ^c^ | | | | |
|  |  | *M* = 0.91  *SD* = 1.04 | *M* = 0.83  *SD* = 0.92 | *X^2^* = 0.147, *df* = 1, *p* = .702 |

*Note*: ^a^ dichotomized variable: medication type received/not received; ^b^ Antidepressants include Tricyclic Antidepressant, Monoamine Oxidase Inhibitor, Noradrenergic Reuptake Inhibitor, Noradrenergic and Specific Serotonergic Antidepressant, Noradrenaline-Dopamine Reuptake Inhibitor, Serotonin Antagonist and Reuptake Inhibitor, Serotonin-Noradrenaline Reuptake Inhibitor, Selective Serotonin Reuptake Enhancer, Selective Serotonin Reuptake Inhibitors, and others (Agomelatine, St. John’s-wort, and ketamine); ^c^ mean change of medication group over seven weeks.

**Table S3**

*Fit indices of latent change score models*

|  | Univariate LCS models | | | Bivariate LCS models | | |
| --- | --- | --- | --- | --- | --- | --- |
| Maladaptive trait | CFI | RMSEA | SRMR | CFI | RMSEA | SRMR |
| Negative affectivity | .986 | .050 | .058 | .969 | .052 | .063 |
| Detachment | .953 | .111 | .083 | .959 | .067 | .078 |
| Antagonism | .959 | .089 | .072 | .981 | .041 | .063 |
| Disinhibition | .987 | .050 | .056 | .982 | .041 | .058 |
| Psychoticism | .967 | .074 | .050 | .939 | .074 | .073 |

*Note*: CFI = comparative fit index; RMSEA = root mean square error of approximation; SRMR = standardized root mean squared residual.

**Table S4**

*Standardized parameters of latent change score models including patients receiving Individual Supportive Therapy*

| Model | Parameter | PID-5-FBF | | | | |
| --- | --- | --- | --- | --- | --- | --- |
|  |  | Negative Affectivity | Detachment | Antagonism | Disinhibition | Psychoticism |
| Bivariate LCS model | Φ | .427 ** | .549 ** | .188 | .531 ** | .414 ** |
|  | γ1 | .045 | .286 * | -.029 | .062 | .174 |
|  | γ2 | -.022 | .058 | -.088 | -.194 | -.031 |
|  | 𝜌 | .539 ** | .688 ** | .125 | .546 ** | .305 * |
| Univariate LCS model | μΔ | -0.677 ** | -0.707 ** | -0.360 * | -0.856 ** | -0.515 ** |

*Note.* Analyses are based on the final sample plus IST condition including n=289 participants. LCS = Latent Change Score. PID-5-FBF = Personality Inventory for DSM-5 Faceted Brief Form; Φ = Depression and maladaptive trait domain correlation at baseline; γ1 = Cross-domain coupling: Trait domain score at baseline predicting rate of change in depression; γ2 = Cross-domain coupling; Depression score at baseline predicting rate of change in trait domain; 𝜌 = Correlated residuals of depression and maladaptive trait domain change scores. μΔ = Intercept of maladaptive trait domain change score * *p* < .01; ** *p* < .001.

**Table S5**

*Results of EffecteLiteR using latent indicator variables*

| Average effect | Conditional effect | Model fit |
| --- | --- | --- |
| Wald *χ2*= 2.80, *df*=1, *p*=.09 | Wald *χ2*= 6.38, *df*=6, *p*=.38 | χ²(66)= 111.861, *p*<.001,CFI=.943, RMSEA=.085, 90%-CI [.057; .112], SRMR=.067 |

* *p* ≤ .01; ** *p* ≤ .001.

**Table S6**

*Regression* *coefficients for the latent model with six covariates and treatment by covariate interactions*

| Coefficient | Variable | Estimate | SE | Est./SE | p-value |
| --- | --- | --- | --- | --- | --- |
| Intercept function g_0_(Z) | | | | | |
| γ_00_ |  | -.25 | .21 | -1.20 | .23 |
| γ_01_ | ξ1 (Z1) | .43 | .16 | 2.60 | .01 |
| γ_02_ | ξ2 (Z2) | -.01 | .16 | -0.06 | .95 |
| γ_03_ | ξ3 (Z3) | .26 | .11 | 2.33 | .02 |
| γ_04_ | ξ4 (Z4) | .13 | .21 | 0.60 | .55 |
| γ_05_ | ξ5 (Z5) | -.04 | .12 | -0.35 | .73 |
| γ_06_ | ξ6 (Z6) | .02 | .14 | 0.14 | .89 |
| Effect function g_1_(Z) | | | | | |
| γ_10_ | X | .42 | .36 | 1.18 | .24 |
| γ_11_ | ξ1 · Z1 | .31 | .31 | 1.02 | .31 |
| γ_12_ | ξ2 · Z2 | -.05 | .23 | -0.23 | .82 |
| γ_13_ | ξ3 · Z3 | -.20 | .18 | -1.09 | .28 |
| γ_14_ | ξ4 · Z4 | -.21 | .30 | -0.69 | .49 |
| γ_15_ | ξ5 · Z5 | -.34 | .23 | -1.51 | .13 |
| γ_16_ | ξ6 · Z6 | .07 | .23 | 0.30 | .77 |

*Note.* Variables contained in the analysis are X (treatment variable, 0 = CBT, 1 = ST, ξ1 = Z1 (pre-treatment BDI-II), ξ2 = Z2 (pre-treatment negative affectivity), ξ3 = Z3 (pre-treatment detachment), ξ4 = Z4 (pre-treatment antagonism) ξ5 = Z5 (pre-treatment disinhibition), ξ6 = Z6 (pre-treatment psychoticism)

**Figure S1: PID domain score distributions by treatment arm at week 0**


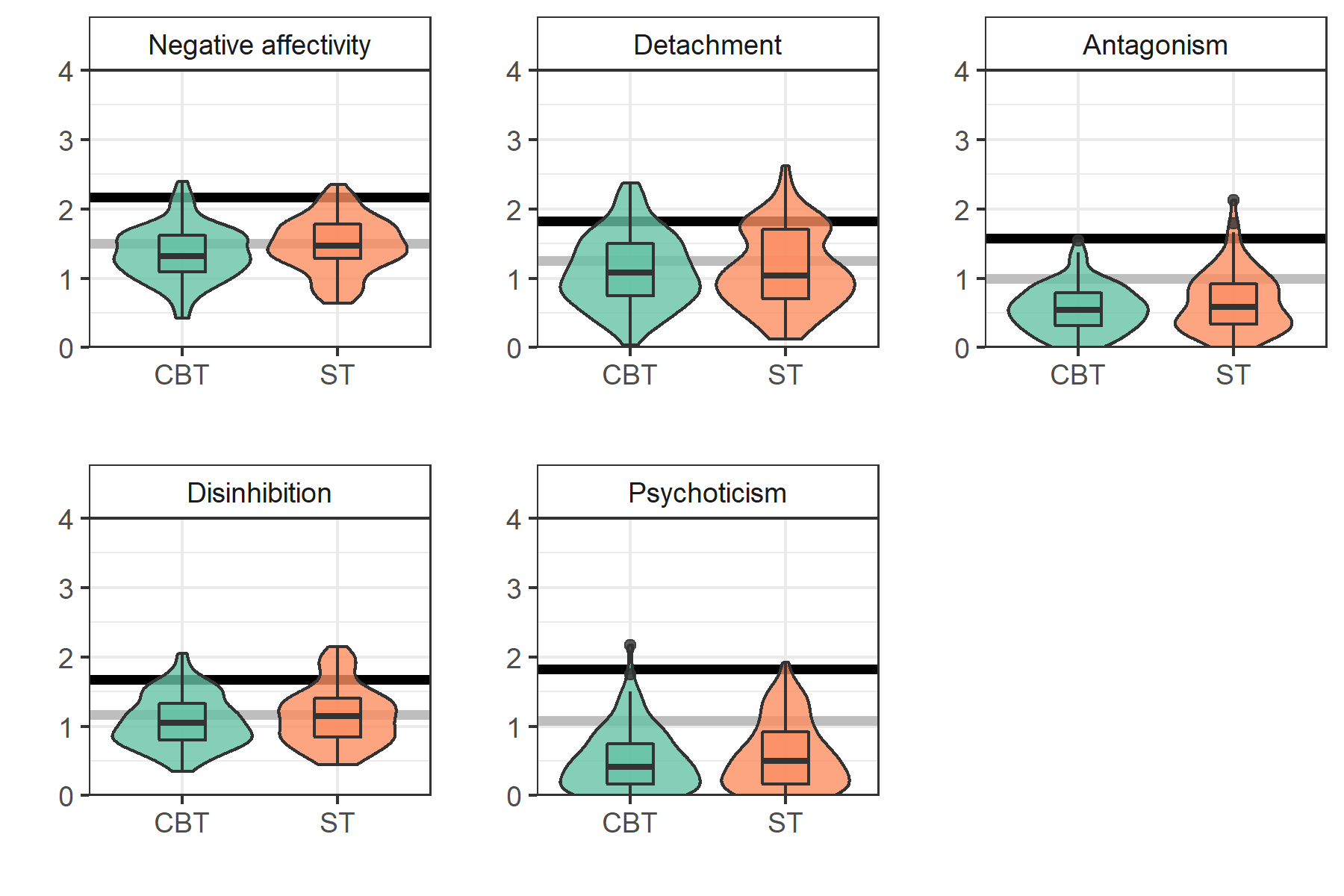


*Note*: Figure shows distributions of PID-5-BFB domain scores (y-axis) per domain and split by treatment. Grey and black horizontal lines show cut-off values for T>60 and T>70, respectively, based on a recent norms provided by Rek *et al.* (2021).

**Figure S2: PID-5-FBF domain score associations across time points and groups.**


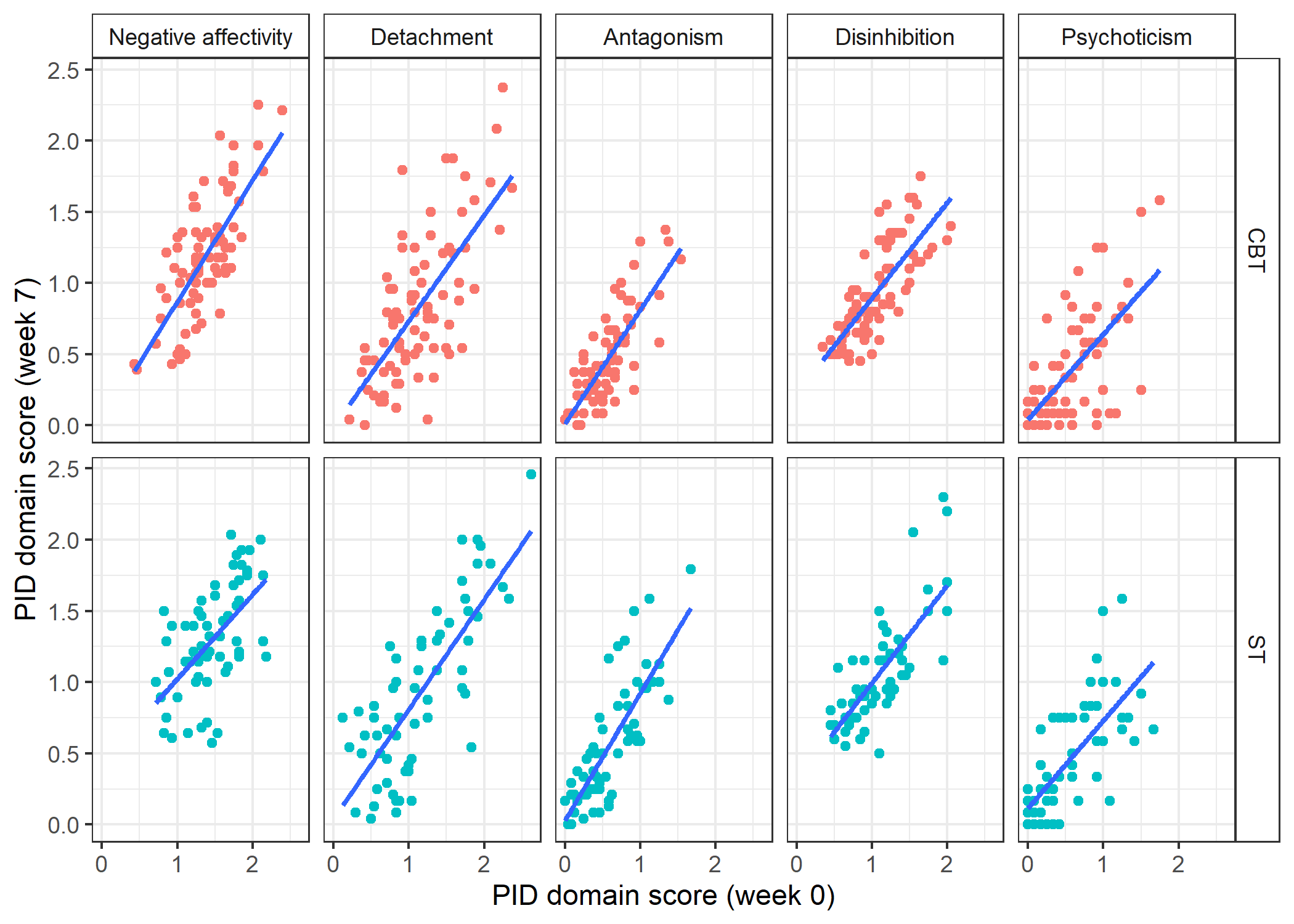


**Figure S3: PID-5-FBF facet score changes over treatment.**

**
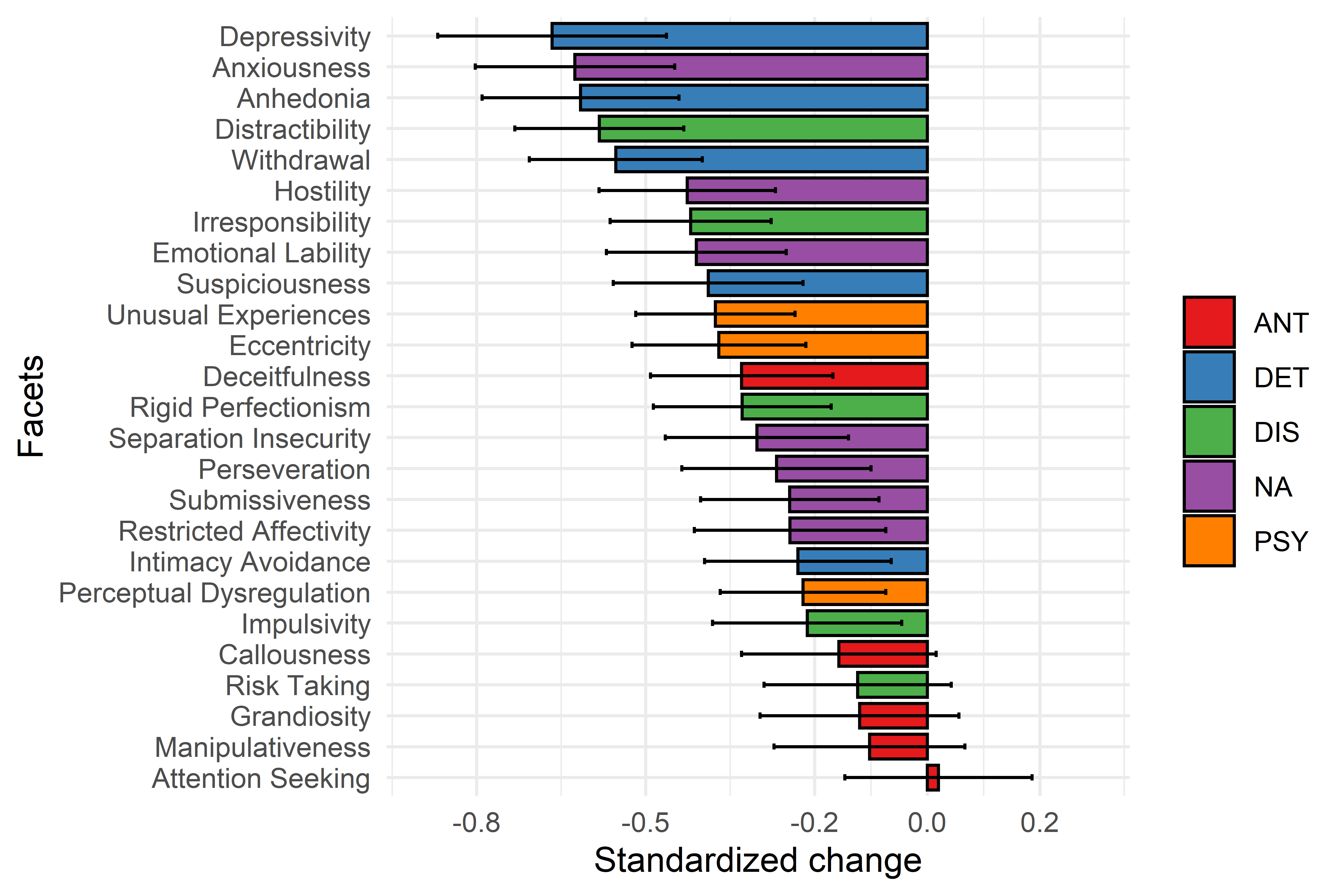
***Note*: This figure shows standardised mean changes in PID-5 FBF facet scores across treatment as estimated using univariate LCS models (with manifest rather than latent variables). Error bars represent 95% confidence intervals for standardised mean change scores. NA=Negative affectivity, DET=Detachment, ANT=Antagonism, DIS=Disinhibition, PSY=Psychoticism.
